# When do young birds disperse? Tests from studies of golden eagles in Scotland

**DOI:** 10.1186/1472-6785-13-42

**Published:** 2013-11-06

**Authors:** Ewan D Weston, D Philip Whitfield, Justin MJ Travis, Xavier Lambin

**Affiliations:** 1 Institute of Biological and Environmental Sciences, Zoology Building, University of Aberdeen, Aberdeen AB24 2TZ, UK; 2 Natural Research, Brathens Business Park, Hill of Brathens, Aberdeenshire, Banchory AB31 4BY, UK

## Abstract

**Background:**

Dispersal comprises three broad stages - departure from the natal or breeding locations, subsequent travel, and settlement. These stages are difficult to measure, and vary considerably between sexes, age classes, individuals and geographically. We used tracking data from 24 golden eagles, fitted with long-lived GPS satellite transmitters as nestlings, which we followed during their first year. We estimated the timing of emigration from natal sites using ten previously published methods. We propose and evaluate two new methods. The first of these uses published ranging distances of parents as a measure of the natal home range, with the requirement that juveniles must exceed it for a minimum of 10 days (a literature-based measure of the maximum time that a juvenile can survive without food from its parents). The second method uses the biggest difference in the proportion of locations inside and outside of the natal home range smoothed over a 30 day period to assign the point of emigration. We used the latter as the standard against which we compared the ten published methods.

**Results:**

The start of golden eagle dispersal occurred from 39 until 250 days after fledging (based on method 12). Previously published methods provided very different estimates of the point of emigration with a general tendency for most to apparently assign it prematurely. By contrast the two methods we proposed provided very similar estimates for the point of emigration that under visual examination appeared to fit the definition of emigration much better.

**Conclusions:**

We have used simple methods to decide when an individual has dispersed - they are rigorous and repeatable. Despite one method requiring much more information, both methods provided robust estimates for when individuals emigrated at the start of natal dispersal. Considerable individual variation in recorded behaviour appears to account for the difficulty capturing the point of emigration and these results demonstrate the potential pitfalls associated with species exhibiting complex dispersal behaviour. We anticipate that coupled with the rapidly increasing availability of tracking data, our new methods will, for at least some species, provide a far simpler and more biologically representative approach to determine the timing of emigration.

## Background

Dispersal is an important but poorly understood behaviour that influences animal population dynamics [1,2]. Dispersal can affect a population’s persistence by linking the components of a spatially structured population’s current or potential distribution, and can affect population expansion through the behaviour of individuals that can exploit resources that are variable across space and time [3]. An understanding of this behaviour is crucial to developing effective conservation strategies. The movement from a natal site or social group to a new site or group where reproduction takes place is termed natal dispersal to distinguish it from subsequent moves to new breeding sites termed breeding dispersal [4]. Three sequential behavioural stages have been identified during natal dispersal; emigration, transience and immigration [5,6], although the terminology varies somewhat across studies including e.g. departure, transience, settlement or start, transfer, stop [2,7-10]. The point of emigration occurs at the start of natal dispersal when individuals depart from the natal environment and enter the transience phase (which can be very short). This event is the first major step in the dispersal process. Identifying the transition between these stages from empirical data remains a challenge, especially as context dependencies and individual strategies can make real movements difficult to decompose in a logical, rigorous and repeatable manner.

Decomposing the life path into its component stages is key to our understanding of dispersal movements [11]. The transitions between the component stages of dispersal are especially interesting as they allow researchers to hypothesise, for example, what determines when an individual emigrates. Identifying the dispersal state of an individual using movement data is vital for understanding how the state of an individual influences its use of habitat and development of movement strategies [12]. In particular where behavioural processes increase in complexity, determining the duration from a transition between behavioural stages becomes increasingly difficult to achieve. This requires the derivation of methods that consistently define the component stages and, therefore, the transition points. In the absence of such methods, empirical studies of dispersal will struggle to realise their full potential. Hence, it is vital that the point of emigration is appropriately and consistently defined.

Many large raptors are relatively long lived, often have a lengthy period of deferred maturity, compete for territories, exploit a wide variety of prey and have a high potential for exploratory behaviour prior to breeding [13]. In keeping with the generic stages derived from research on several taxa, studies of natal dispersal in raptors (birds of prey) have focussed on four key developmental stages: 1) a post-fledging dependence period ending in emigration from the natal environment; 2) a long transitional phase (often termed “juvenile dispersal”, synonymous with transience); 3) provisional settlement in temporary settlement areas, where individuals establish more or less stable home ranges; and 4) settlement at a breeding site [10,14-25].

As the timing of the phases of juvenile dispersal can indicate some of the causes of variation between individual strategies there is a general need to develop reliable methods to identify when these changes occur. Two recent studies have aimed to compare several methods for estimating the timing of the start of natal dispersal [20,21]. Generically, existing methods fall under two main categories: 1) distance threshold, and 2) displacement rate based methods. These two studies highlighted that there was a tendency to assign prematurely the point of emigration across these methods. Furthermore, there was a tendency for these methods to yield inconsistent estimates for individuals that used complex strategies, in particular those that engaged in a variable number and timing of pre-dispersal excursions. So it would follow that the inter-individual variation in the prevalence of pre-dispersal excursions violates the assumptions of the assignment methods used. Whereas this may not be problematic for some applications, e.g. comparing geographical differences in timing of emigration, it is important to identify the timing of transition from dependence to independence from the natal locus when studying dispersal. Thus, the objectives of this study are to use novel data collected in Scotland on the golden eagle *Aquila chrysaetos* - a species notorious for its high inter-individual variation in dispersal strategy: 1) to provide an objective re-assessment of existing methods to estimate the point of emigration at the start of natal dispersal; and 2) to evaluate the value of a novel method incorporating GIS predicted breeder home range boundaries as a proxy for the area of potential parental influence and a minimum time away from the natal home range as a proxy for independence from potential parental feeding.

## Methods

### Geographic area

Nestlings were sampled from eight biogeographic regions following the same division of regions used in other recent golden eagle studies in Scotland [26,27] (Additional file 1). As such this encompassed a large amount of the variation in habitat occupied by golden eagles in Scotland.

### Satellite tagging and tracking

Under appropriate legislative licences from the British Trust For Ornithology for fitting satellite transmitters and from Scottish Natural Heritage for visiting nests for the purpose of fitting transmitters, 24 golden eagles were fitted with transmitters from 20 different natal home ranges (2007 n = 1, 2008 n = 5, 2009 n = 4, 2010 n = 14); including three home ranges where chicks were fitted with transmitters in different years and two broods where two chicks were fitted with transmitters in the same year. Nests were visited to fit transmitters when the chicks were between approximately 45-70 days old, based on plumage [28]. All transmitters were fitted using 13 mm Teflon Ribbon (Bally Ribbon Mills, Bally, Pennsylvania). Eagles were fitted with breakaway harnesses stitched with either cotton or linen thread at the central point over the sternum [29,30]. Two different transmitter models were used:

1) Battery powered 105 g Argos/GPS tags from Microwave Telemetry Inc. (n = 14).

2) Solar powered 70 g Argos/GPS tags from Microwave Telemetry Inc. (n = 10).

Golden eagles weighed between 3.4 and 5.0 kg at time of tagging and transmitter weights were, in all cases, less than the 3% recommended maximum of body weight [30].

Transmitters sent GPS locations that were collected at intervals ranging from 1 per hour to 1 per day, depending on the transmitter model and programmed duty cycle. Only locations within 365 days of fledging were included (22,954 GPS locations). To maintain a constant temporal scale all the GPS fixes were filtered to maintain a single location per day per transmitter at 12 h. Where locations at 12 h were absent the nearest fix on that day was taken with the preference for fixes after 12 h (retaining 7,759 GPS locations). All data was transmitted via the Argos satellite tracking systems [31].

Approval of licenses is to an individual and a year so with multiple individuals and years there are several license numbers.

### The start of dispersal

The point of emigration at the start of natal dispersal in individual birds was estimated using twelve methods (Table 1). The methods 1-11 fall into two distinct categories:

a) Methods that use a derived distance-from-natal-nest threshold (methods 1-7) with or without an additional restriction of a minimum duration that this distance has to be exceeded before dispersal is deemed to have started [16,21,32,33]. Distance thresholds have been derived on a population level from information about the distance between nests (for territorial species), or derived by simple visual examination of the spatio-temporal patterns in the data by the researcher [20,21]. Few attempts have been made to set this at an individual level, but Delgado & Penteriani [10] derived their distance threshold based on the mean distance from the nest an individual was recorded and classified the point of emigration as occurring when all subsequent records were greater than the mean. This group of methods assumes that the defined distance thresholds robustly reflect both an appropriate distance and duration to distinguish emigration from other behaviours. As such it can be less flexible to individuals travelling further than this prior to emigration or passing within this distance after the point of emigration, a phenomenon which is commonly observed across several taxa as part of the dispersal process; e.g. [17,20,33-40].

b) Methods that utilise the maximum rate of increase in distance from the nest as described by the coefficient of variation (Methods 8-11); the ratio of standard deviation to the mean over a set duration or number of locations [20,21]. The number of locations used or the number of days in the window can be altered to reflect some biological understanding of the movement trajectory. In general, increasing the number of locations over which this is calculated increases the smoothness of the resulting change in coefficient of variation (inferring a reliance on the number of records – imposed by the limitations of different telemetric technologies). This method assumes that the point of emigration at the start of dispersal is the most distinct movement away from the natal home range, thus the variation in distance (standard deviation) is largest compared to the mean distance from the nest at the point of emigration. In essence they are statistical methods that generate a coefficient of variation that describe rate of movement away from the nest over time and assumes that the start of dispersal is when the rate of movement away from the nest is greatest.

**Table 1:** Methods for estimating the timing of dispersal in juvenile raptors (emigration from the natal home range and independence from parental resources)

**Method**	**Description**	**Reference**
1	First day beyond circular parental territory radius (half the mean nearest neighbour distance) – 2.9 km	[20,21]
2	First day beyond the mean nearest neighbour distance – 5.8 km	[20,21]
3	First day over 20 km from natal nest	[16,20]
4	First day beyond circular parental territory radius (half the inter-nest distance) and not within that distance for 2 consecutive locations	[17,20,21]
5	First day beyond the mean inter-nest distance and not within that distance for 2 consecutive locations	[20,21]
6	All locations over the mean distance to nest	[10]
7	First day beyond maximum ranging distance (9 km) and not within usual range (6 km) for the following 10 days	This study
8	Highest coefficient of variation over a 3 record period	[20,21]
9	Highest coefficient of variation over a 5 record period	[20,21]
10	Highest coefficient of variation over a 10 record	[20,21]
11	Highest coefficient of variation over a 30 day period	[20,21]
12	Maximum change in proportion of locations inside natal home range (-30 days: +30 days )	This study

Methods 1-7 are distance threshold methods and 8-11 are coefficient of variation calculated around each day.

Methods 1-6 and 8-11 have been previously used but, as alluded to above, there are some potential problems with these methods identifying a point of emigration that does not fit the observed location pattern. They particularly vary in their suitability for individuals making pre-dispersal excursions and make assumptions about their duration or the rate of movement from the natal locus and its relation to the point of emigration. Therefore, we generated a method (7: Table 1) also using a simple distance threshold based on a biological surrogate of expected movement distances and maximum excursion duration of juveniles. This method (7: Table 1, see also [28]) assumes that dispersal had occurred if a golden eagle was recorded over 9 km from the natal nest and did not return to within 6 km of the natal nest for the following ten days. These distances were based on a study of radio-tagged breeders in mainland western Scotland, where individuals were recorded up to 9 km from their nest sites but over 98% of observations were within 6 km of the nest [41]. It is known that breeders in other areas, where breeding densities are higher, do not range as far [42,43] and so the selected values for this population-wide metric for distance thresholds may be slightly excessive. Our choice of a ten day return threshold was based on the maximum time that we judged young golden eagles could survive without food from a parent, based on information in Watson [28], and under the assumption that young eagles surviving without any parental provisioning are independent and therefore potentially undergoing dispersal.

**Figure 1:**
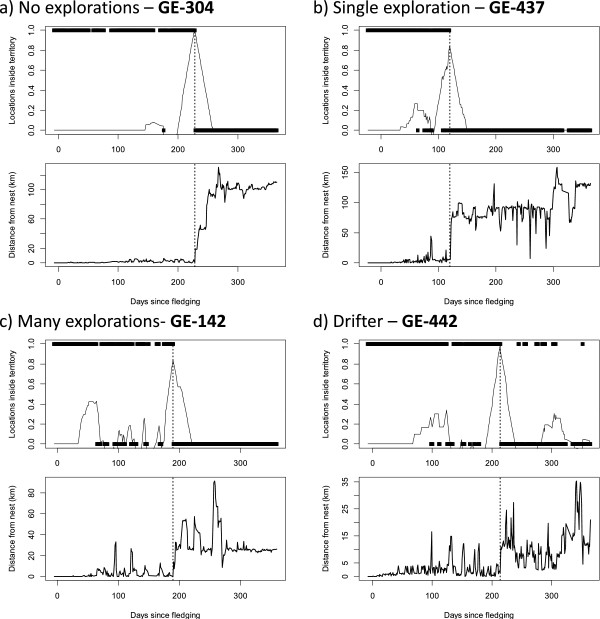
**Determining the point of emigration for a range of illustrative eagle behaviours (see Table**1**).** The vertical dotted line denotes on all panels the calculated timing of dispersal using Method 12. Upper panel: points - occupation of natal home range (natal home ranges defined using the PAT model of golden eagle home ranges) at each time point (1 = in natal home range, 0 = outside of natal home range); solid line – _*diff*_*P* see Methods for details of calculation. Lower panel: solid line – distance from the nest. Different observed emigration behaviour; **a)** no pre-dispersal excursions, **b)** single pre-dispersal excursion, **c)** many pre-dispersal excursions, and **d)** drifter. For the full range of individuals see Additional file 2.

**Figure 2:**
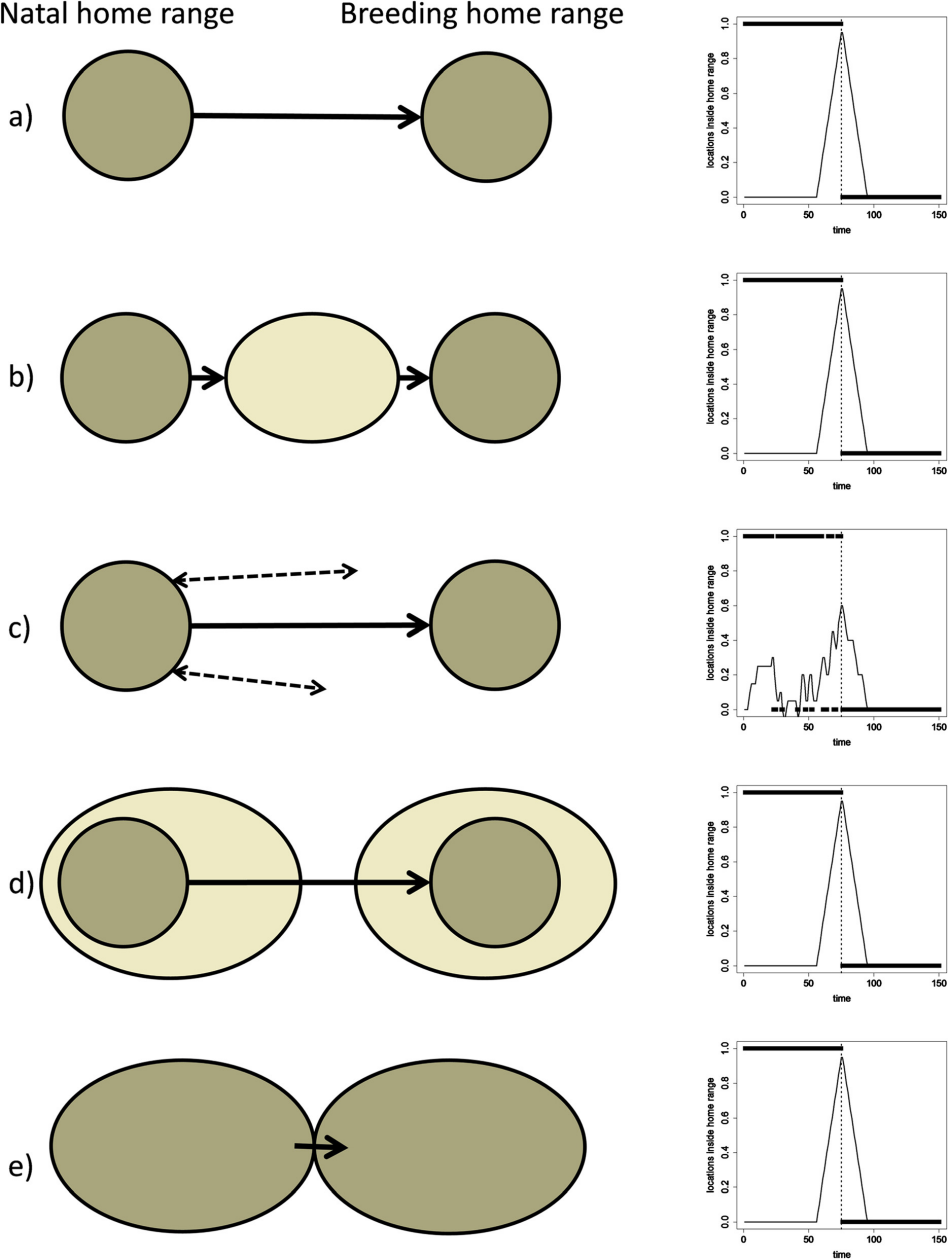
**Illustrative representation of several natal dispersal processes and comparative graphical representation of the estimated point of dispersal from simulated data calculated using method 12.** Left panels are pictographic representations of **a)** natal dispersal of an individual emigrating and settling in a single movement from the natal home range to its own breeding home range, **b)** natal dispersal where emigration is a single movement away from the natal home range followed by the formation of a transience home range prior to a single movement to its own breeding home range, **c)** natal dispersal where emigration is preceded by prospecting forays and emigration is a single movement to its own breeding home range, **d)** natal dispersal of a seasonally territorial species where an individual uses an extended home range outside of the breeding season, overlapping with the natal home range before making a distinct movement to a new area that contracts during the territorial season into its own breeding home range, **e)** natal dispersal with a distinct movement between one social group’s home range and a new social group’s home range. Right panels are a graphical representation of simulated data from an individual from each corresponding 5 strategies. All individuals emigrate after 75 time units of a 150 time unit long follow. Points - occupation of natal home range at each time point (1 = in natal home range, 0 = outside of natal home range), solid line – _*diff*_*P* see Methods for details of calculation (using 20 day sliding window), vertical dotted line – time = 75 and individuals emigrate.

Method 12 is novel to the present study and based on classifying an individual as inside or outside of its parents’ home range in order to objectively identify when an individual becomes independent. It is the most data-intensive and in developing this second novel method (12: Table 1), we wished to address some of the difficulties that have been identified in previous studies identifying the point of emigration at the start of natal dispersal in raptors [20,21]. While method 7 was built on a simple population wide distance and duration threshold, method 12 was designed to incorporate a two-dimensional representation of the likely parental home range for each juvenile eagle, such that the extent and shape is determined by the distribution of neighbouring nests and expected ranging distances. This method therefore relies on individual-centric measures of parental home range boundaries for which we have used the truncated Thiessen polygon based PAT (Predicting Aquila Territories) model [42]. We used the current nest site in the year of tagging instead of the mean location for the last 10 years [42] as Fielding & Haworth [44] found it provided a better estimate of adult (parental) range use within a year, and also represents the natal nest site for a young eagle in that year. The following procedure was carried out following McLeod *et al.*[42]: 1) identify the focal home range centres based on the natal nest sites occupied that year; 2) identify neighbouring home range centres within 12 km buffer of the natal nest site, either from local monitoring records or if unavailable, from 2003 national census data [45], 3) create range boundaries between pairs by Dirichlet tessellation to create Thiessen polygons, which were initially truncated at 6 km from the nest, 4) calculate area of 6 km truncated Thiessen polygon and use equation in McLeod *et al.*[42] to predict the expected maximum ranging distance, 5) create Thiessen polygons that are truncated at the calculated maximum ranging distance for each home range. The home range boundaries were created in ArcMap® 9.3 (ESRI). The method is similar to methods 8-11 that use statistical metrics as it is similarly based on identifying the time the maximum rate of change occurred, but using the rate at which the parents’ home range is visited as the variable rather than just the change in distance from the nest. Each GPS location was assigned to either inside the parents’ home range or outside of it based on the truncated Thiessen polygons. As dispersal is a process that occurs on a large temporal scale, the proportion of locations inside the natal home range for 30 days before (*P*_*1*_) and 30 days after (*P*_*2*_) for each record (_*diff*_*P = P*_*1*_ - *P*_*2*_) was calculated to capture changes in use across a two month sliding window. The point of emigration was calculated as the point at which individuals had the largest difference (_*max*_*P = maximum*_*diff*_*P*) between these two values (Figure 1). This method requires the most information to implement and was commensurate with our definition of the point of emigration at the start of natal dispersal for several illustrative natal dispersal processes (Figure 2). As this method was accurate for the natal dispersal processes tested and under close examination was observed to be robust for individuals illustrative of the variation in behaviour within our study (Figure 1, all examples in Additional file 2), we used it as a reference method when comparing the other 11 methods that required less information to implement.

We assigned the individual date of fledging based upon GPS fixes during the first weeks after tagging. We did this on a subjective basis due to the wide scatter of GPS fixes in relation to the actual nest site (mean ± standard deviation 46 ± 58 m) as a result of interference by structures around the nest such as cliffs and tree canopy.

### Statistical analysis

We used method 12 as the “reference” or “benchmark” method against which to evaluate the other 11 methods as it was the most ‘data intensive’ method, by way of local information on parental range use and number of GPS fixes of juvenile movements. To quantitatively assess which method most closely matched the reference values from method 12, we calculated the mean deviation $\sqrt{\Sigma {\left(\mathrm{Residual}\right)}^{2}/n}$ and the mean bias *ΣResidual/n* of each method from method 12 on an individual by individual basis. As such, the methods applicability to our study system could be assessed in terms of both the overall deviation from the expected point of emigration (as estimated by method 12) and the overall direction of the bias (negative values underestimate and positive values overestimate). Statistical analysis was carried out using R 2.15.0.

## Results

The home range sizes obtained from the PAT model for golden eagles provided an estimate of home range size that varied from home range to home range (median 100.5 km^2^; range 32-113 km^2^). A total of 8% (283 of 3483) of locations were outside of the modelled natal home ranges, up to a maximum distance of 44 km from the natal nest prior to the point of emigration as estimated by method 12. Two individuals were never recorded outside of the natal home range prior to their estimated point of emigration. Twenty-two out of 24 individuals re-entered their natal home range after having been deemed to have dispersed and this amounted to 1.8% of locations between the point of emigration and 365 days after fledging (80 locations of 4276; median distance 2.6 km range 0.15-5.7 km).

**Figure 3:**
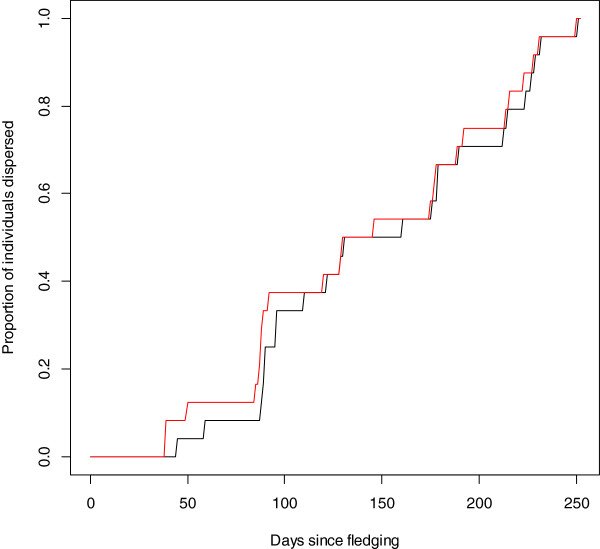
**Percentage of golden eagles (n = 24) dispersed from their natal home ranges as calculated by the two best methods.** Method 12 (black line): r^2^ = 0.98; Method 7 (red line): r^2^ = 0.97.

The method used to calculate the point of emigration had a significant effect on the estimated values for each individual (Friedman rank sum test χ^2^ = 168.20, df = 11, p < 0.001). The range of estimates of the point of emigration for all the methods, was 14 to 365 days after fledging, encompassing almost the entire period of our study (Table 2). According to our reference method, the point of emigration occurred over a wide window (39-250 days after fledging - method 12). This occurred at a relatively continuous rate between 39 and 250 days (Figure 3). On a population level, methods 3, 5 and 7 were not significantly different from method 12 (Wilcoxon pairwise comparisons: method 3 - W = 287, p = 0.99; method 5 - W = 245, p = 0.38; method 7 W = 318, p = 0.53). Methods 1, 2, 4 and 6 were significantly different and methods 8, 9, 10 and 11 were significant at the 5% level but not after Bonferroni corrections for multiple comparisons (Wilcoxon pairwise comparisons - significance level after Bonferroni correction = 0.0045: method 1 - W = 64, p < 0.001; W = 145,method 2 - p = 0.003; method 4 - W = 115, p < 0.001; method 6 - W = 514, p = <0.001; method 8 - W = 171, p = 0.016; method 9 - W = 184, p = 0.032; method 10 - W = 159, p = 0.008; method 11 - W = 151, p = 0.005). Method 7 had the lowest mean deviation from method 12 (mean deviation = 12) followed by method 5 (mean deviation = 35), which in real terms relates to an average of 12 and 35 days between the estimate for method 12 and each of these methods, considerably better than the average across all methods (77 days). Method 3 has the lowest overall bias (bias = -4) followed by method 7 (bias = 7).

**Table 2:** **Estimated timing of the point of emigration in days since fledging calculated by 12 methods (Table**1**)**

**Method**		**Median (Range)**	**Bias**	**Mean deviation**
1	First day beyond 2.9 km	42 (22–119)	−90	109
2	First day beyond 5.8 km	69 (40–208)	−56	77
3	First day beyond 20 km	103 (52–267)	−4	44
4	First day beyond 2.9 km for 2 consecutive locations	68 (40–163)	−64	82
5	First day beyond 5.8 km for 2 consecutive locations	87 (45–234)	−18	35
6	First day when all subsequent locations are over the mean distance to the nest.	283 (88–365)	134	172
7	First day beyond 9 km and not within 6 km for the following 10 days	145 (45–251)	7	12
8	Highest coefficient of variation over a 3 record period	67 (24–239)	−47	68
9	Highest coefficient of variation over a 5 record period	68 (14–240)	−43	78
10	Highest coefficient of variation over a 10 record period	69 (14–243)	−53	86
11	Highest coefficient of variation over a 30 day period	66 (14–236)	−56	88
12	Maximum change in proportion of locations inside natal home range (−30 days: +30 days )	144 (39–250)		

Method 7 provided the best fit with the reference estimates provided by method 12 (Table 2; Figure 3). The distance threshold methods (1 – 7) on the whole produced closer estimates to the evaluation method than did the coefficient of variation methods (8 – 11) (Table 2). Within the range of values tested, increasing the threshold distances that an individual had to move away from their natal nest in order to be considered as having dispersed improved the estimates, as did increasing the duration over which the coefficient of variation was calculated, but only slightly (Table 2). There was a general tendency for most of the methods (with the exception of method 6 & 7) to provide earlier estimates of the point of emigration at the start of natal dispersal than the reference technique of method 12 (Table 2).

## Discussion

The start of natal dispersal in our population of golden eagles took place over a hugely variable period of time, with all individuals emigrating from their natal home range within their first year. Based on our best estimates (method 12) the first individual started to disperse just 39 days after fledging and the rate of departure was constant across our sample until the last individual left its natal home range 250 days after fledging. Although all individuals departed their natal home ranges within their first year, this period represents only a small fraction, ~ 25% relative to the “dispersal lifespan” *sensu*[46] of approximately 3-5 years of transience [28] that are thought to separate emigration from the natal range and settlement in a breeding site.

Twenty-two of the 24 golden eagles ventured out of their natal home ranges prior to their emigration at the start of natal dispersal. These trips took them up to 44 km from their natal nests. These pre-dispersal excursions are similar to that found in a number of raptors, including common buzzard *Buteo buteo*, Bonelli’s eagle *Aquila fasciata,* Spanish imperial eagle *Aquila adalberti* and northern goshawk *Accipiter gentilis*[17,20,33,34]. Similar behaviours have been reported in a number of other species including; red-bellied woodpeckers *Melanerpes carolinus*[35]*,* red squirrel *Tamiasciurus hudsonicus*[36,37], spotted hyena *Crocuta crocuta*[38]*,* black bear *Ursus americanus*[39] and white-tailed deer *Odocoileus virginianus*[40]. Whilst this behaviour is an integral part of the dispersal process, under our definition it is not the point of emigration at the start of natal dispersal. Yet as a prospecting mechanism, pre-dispersal excursions are likely to be useful for potential dispersers to assess the competitive environment in order to decide when to disperse or where to disperse to, and in some species may be the mechanism to decide if they disperse at all.

The variation between methods of estimating the point of emigration for golden eagles was substantial and reflects the high inter-individual variation in timing and complexity of behaviour during early life. Whilst some of the methods provided similar estimates of the spread of dispersal timing at a population level there was a general trend, with the exception of methods 6 & 7, to estimate the timing of dispersal as being much earlier than method 12′s estimates, presumably because pre-dispersal excursions lead to premature assignment of the point of emigration.

Method 12, as expected given its data-intensive composition and, hence, classing it as the ‘benchmark’ method, appeared to cope well with the variability in behavioural strategies, particularly pre-dispersal excursions and rapid long movements not occurring at the start of dispersal, because it used smoothing over a relatively long period (30 days) and only assessed the point of emigration based on presence or absence from the natal home range. As we observed that birds did not appear to disperse until a minimum of 40 days after fledging this allowed us to use a 30 day smoother and still detect the point of emigration. The main requirement for this method is to distinguish between parental influence (our predicted home ranges) and non-parental influence (everything outside of the natal home ranges) in our situation based on an individual’s location. In the absence of any site-specific studies that allowed observational definition of such boundaries e.g. Cox & Kesler [35] for red-bellied woodpeckers, we used the PAT [42] to infer the natal home range boundaries as it provides the best available prediction of golden eagle range use both in Scotland [42] and elsewhere [47], only requiring the locations of other breeding eagles within 12 km from the focal site to estimate boundaries. This process could easily be adjusted when working in areas with less precise information, an approximate expected range size and the locations nearest neighbours within double the expected range radius. While method 12 is analogous to the distance threshold methods for home ranges without near neighbours it also provides a solution where home ranges are of uneven size and shape due to shared boundaries.

For territorial species, a surrogate for natal influence could be any area conforming to home range concept of the parents; from a simple Dirichlet tessellation if the home ranges are contiguous, minimum convex polygon, or utilisation distribution kernel from parental observations [48-51]. The method could also be applied to a social group and the change in association with the natal group to another. Conceptually this method provides a high degree of biological realism by conforming to the definition that dispersal is the movement from a natal group or site to a new group or site where breeding may take place if an individual survives [52], and thus could be adjusted to deal with other strategies across many different taxa so long as they conform to this broad definition. Dirichlet tessellation, as used directly in method 12 to estimate parental home range boundaries is widely applicable across many taxa and has been used over many years [53-55].

We found the golden eagle in Scotland to exhibit complex behaviours that made it difficult to apply previously published methods, despite altering threshold values to fit local values. The generic difficulty assigning the start of natal dispersal was suggested by Soutullo *et al.*[56], who suggested two methods to use in future studies yet they differed on an individual level by as much as 78 days and on average by 20 days. Within the distance thresholds the main failures of methods tested by our study were: 1) individuals often travelled further than is tolerated by the thresholds prior to dispersal; and 2) the additional condition of a certain number of locations over this distance that had to be met before an individual was deemed to have dispersed was too small in all but method 7 and method 6. This was probably due to the duration of some temporary departures from the natal site prior to dispersal and that some studies under other methods based departure more on the availability of tracking data rather than based on biological constructs of temporal independence. The tendency of these methods to prematurely assign the point of emigration due to pre-dispersal excursions has been noted in several other studies amongst other birds of prey [18,20,33]. Method 6 failed to provide a useful threshold set at an individual level due to the less settled movement strategies of our study species compared to that of the species it was originally used on [10], in particular golden eagles ranged widely and returned often very close to the natal nest during the early transience phase. Within the coefficient of variation methods, based on the rate of movement away from the natal site, the main failure was that individuals could undertake quick and long movements away from the natal home range and/or drift away at the point of dispersal. As this group of methods is also rate-based, the scale of the movements are not taken into account such that an individual that was located very close to the nest and then subsequently further away would show a large change in rate due to the scale on which the displacement occurs. In spite of these difficulties, method 7 provided a very good population wide metric to describe the point of emigration without the need to take variation in home range size into account.

Despite the pre-dispersal excursions from the natal home range and ontogenic jumps in movement there is still considerable scope to apply a rigorous definition of dispersal to tracking data. It is true that, for many species, lack of data may constrain the potential to use these new methods. However, data are becoming readily more available, tracking devices lighter and more sophisticated, and tracking locations less expensive to collect. While we have presented data from a species of large raptor that exemplifies a particular problem, these difficulties are likely to occur in other taxa with analogous movement strategies. As there is a wide variety of general dispersal mechanisms [57] any methods used should be appropriate to the strategy and thus while a simple method may work for a simple strategy as individuals start to display more complicated behaviours some systematic biases may occur in association with particular behaviours. These complex strategies are an integral part of an individual’s life history and are likely to be important to furthering our understanding of the dispersal process as a whole. Although we are unlikely to be able to irrefutably pinpoint when an individual emigrates at the start of natal dispersal from tracking data alone it is important to develop methods that can accommodate species and individuals with more complex patterns, and in this way get us closer to the true point at which an individual emigrates. Capturing the biological realism of the processes we are studying can help us to create relatively simple methods that allow us to do this, in spite of high inter-individual variation. In this respect, our study indicates that if the start of dispersal is to be consistently estimated, then it needs to be based on the behaviour of individuals, the environmental context of such individual behaviour and, hence, methods that can cope with documenting such individual variability. Such methods are not especially onerous or restricted to large raptors, as in our study species. An important message of our study is that consistent estimates need to be based on biology (so that estimates are not due to methodological rather than biological factors) and that studies attempting to document the start of dispersal need to be individually-based and, hence, be supported by data that allow such individuality to be estimated.

## Conclusions

Dispersal is a key behavioural process with implications for how individuals distribute themselves throughout the environment. Although the dispersal process can be distilled into a common framework of emigration, transience and immigration the actual behavioural mechanisms for dispersal vary greatly. As strategies get more complex it can become difficult to decompose an individual’s movement path into its component stages. We used previously published methods alongside two we have derived to calculate the point of emigration of 24 golden eagles. We took one of our new methods to be the reference. Method 12 seemed to adequately reflect the natal dispersal process both within the variation found in our study and across several simulated example strategies, but relied on a realistic representation of the natal environment by way of the most data-intensive composition, and used it to evaluate the other 11 methods. We found that due to the complexity of movements in their first year of life that golden eagles in Scotland were particularly difficult to assign a dispersal date to. Previously published methods did not perform as well as they perhaps had on other species, probably as a result of pre-dispersal excursions undertaken by juveniles prior to the emigrating and high mobility during the transience phase. The two new methods were better able to cope with this behaviour. We suggest that, like many other processes that influence populations, the start of natal dispersal is individualistic. Defining its onset therefore needs to be based on the behaviour of individuals and, hence, on data that allow individuals’ behaviour to be accounted for.

Our analysis illustrates how difficult it can be to identify in a rigorous and repeatable manner when an individual initiates dispersal, indeed our two preferred methods (methods 7 and 12) were on average 12 days different. We found our two simple methods could be used to dissect an individual’s movement path at the point of emigration as individuals started natal dispersal. With the current interest in dispersal and increasing number of studies tracking potential dispersers there is now scope to apply a simple approach to analysing complex dispersal movements. This is of particular importance as decomposing complex dispersal movements is key to furthering our understanding of dispersal strategies.

## Competing interests

The authors declare that they have no competing interests.

## Authors’ contributions

EDW designed the study, new methods, ran the analysis and wrote the manuscript. DPW, XL and JMJT provided comments and improvements throughout the analysis and preparation of the manuscript. All authors have read and approved this manuscript. XL was supported in part by a Leverhulme Trust fellowship and grants from NERC. EDW was funded by a University of Aberdeen and Natural Research Ltd co-funded studentship.

## Supplementary Material

Additional file 1 Table of tagging regions. Click here for file

Additional file 2 The point of emigration for all 24 individuals using method 12. Click here for file
