# Supplementary material for: When do young birds disperse? Tests from studies of golden eagles in Scotland
Source: BMC Ecol. 2013 Nov 6;13:42. doi: 10.1186/1472-6785-13-42 (PMC3833264; doi:10.1186/1472-6785-13-42)
Supplement: Additional file 1 — Table of tagging regions. [file 1472-6785-13-42-S1.pdf]

Supplementary table 1. Details of the year, territory, biogeographic zone [from 26, 28] and transmitter type of 24 golden eagles fitted with GPS satellite transmitters in Scotland.

Individual	Year	Home range	Brood size	Biogeographic zone	Transmitter type
GE-301	2007	Bre-2	1	Breadalbane and East Argyll	GPS (battery)
GE-166	2008	Arg-1	2	Argyll West and Islands	GPS (battery)
GE-169	2008	Bre-1	1	Breadalbane and East Argyll	GPS (battery)
GE-132	2008	Cai-1	1	Cairngorm Massif	GPS (solar)
GE-167	2008	Cai-6	1	Cairngorm Massif	GPS (battery)
GE-170	2008	Cai-7	1	Cairngorm Massif	GPS (battery)
GE-437	2009	Arg-3	1	Argyll West and Islands	GPS (battery)
GE-440	2009	Cai-4	2	Cairngorm Massif	GPS (battery)
GE-441	2009	Nws-1	1	North West Seaboard	GPS (battery)
GE-442	2009	Wes-1	1	Western Isles	GPS (battery)
GE-856	2010	Arg-1	2	Argyll West and Islands	GPS (battery)
GE-304	2010	Arg-2	1	Argyll West and Islands	GPS (battery)
GE-109	2010	Cai-2	1	Cairngorm Massif	GPS (solar)
GE-111	2010	Cai-3	2	Cairngorm Massif	GPS (solar)
GE-382	2010	Cai-5	1	Cairngorm Massif	GPS (solar)
GE-115	2010	Cai-7	2	Cairngorm Massif	GPS (solar)
GE-124	2010	Cai-8	2*	Cairngorm Massif	GPS (solar)

GE-139	2010	Cai-8	2*	Cairngorm Massif	GPS (solar)
GE-142	2010	Cen-1	1	Central Highlands	GPS (solar)
GE-857	2010	Noh-1	1	Northern Highlands	GPS (battery)
GE-858	2010	Pea-1	1	Peatlands of Caithness and Sutherland	GPS (battery)
GE-106	2010	Pea-2	2*	Peatlands of Caithness and Sutherland	GPS (solar)
GE-107	2010	Pea-2	2*	Peatlands of Caithness and Sutherland	GPS (solar)
GE-867	2010	Wes-2	2	Western Isles	GPS (battery)

* Broods where more than one chick was tracked
