# Supplementary material for: When do young birds disperse? Tests from studies of golden eagles in Scotland
Source: BMC Ecol. 2013 Nov 6;13:42. doi: 10.1186/1472-6785-13-42 (PMC3833264; doi:10.1186/1472-6785-13-42)
Supplement: Additional file 2 — The point of emigration for all 24 individuals using method 12. [file 1472-6785-13-42-S2.pdf]

GE-301

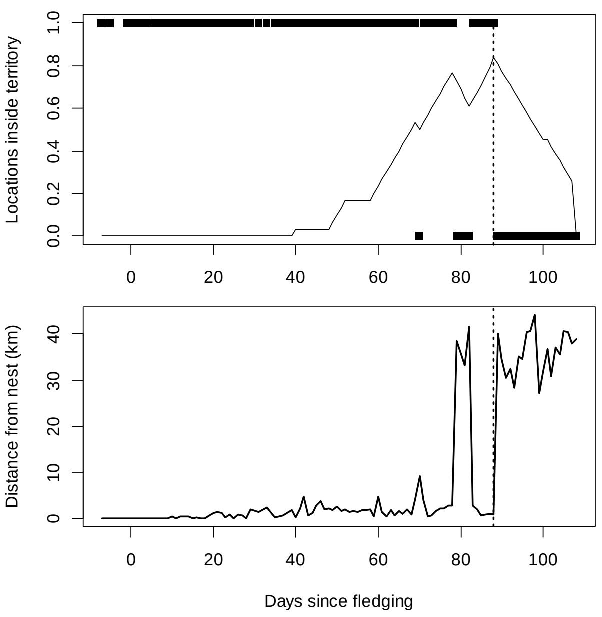

GE-166

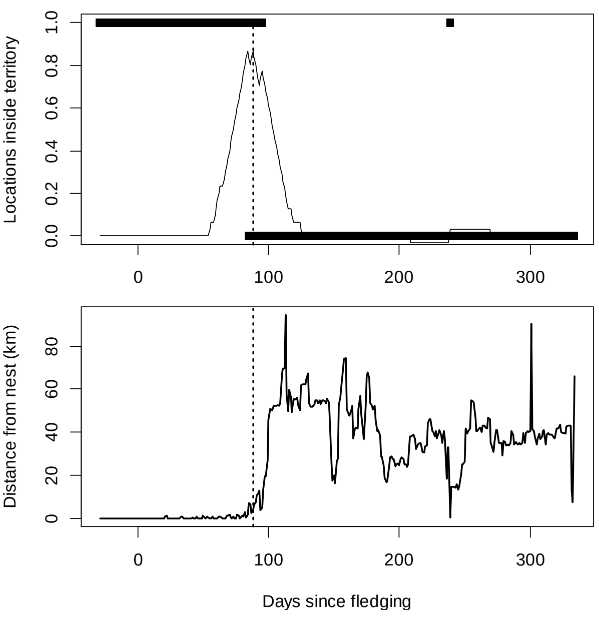

GE-169

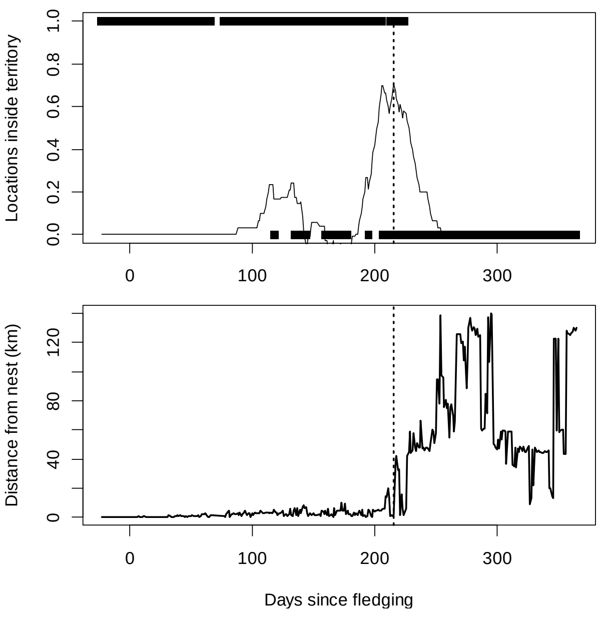

GE-132

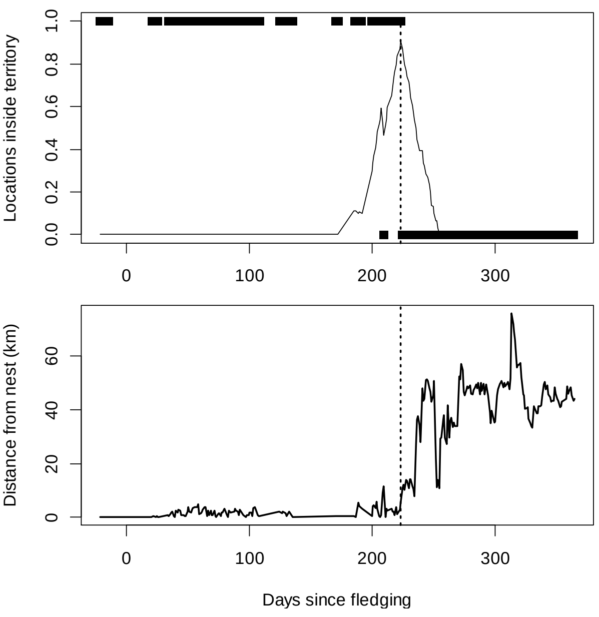

GE-167

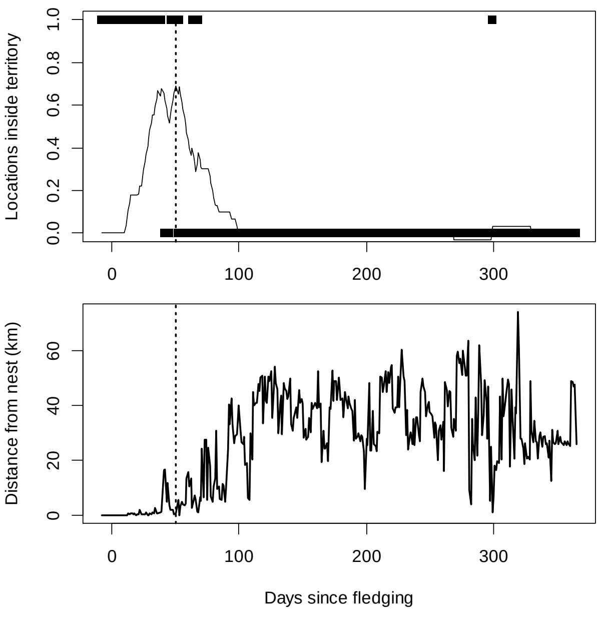

GE-170

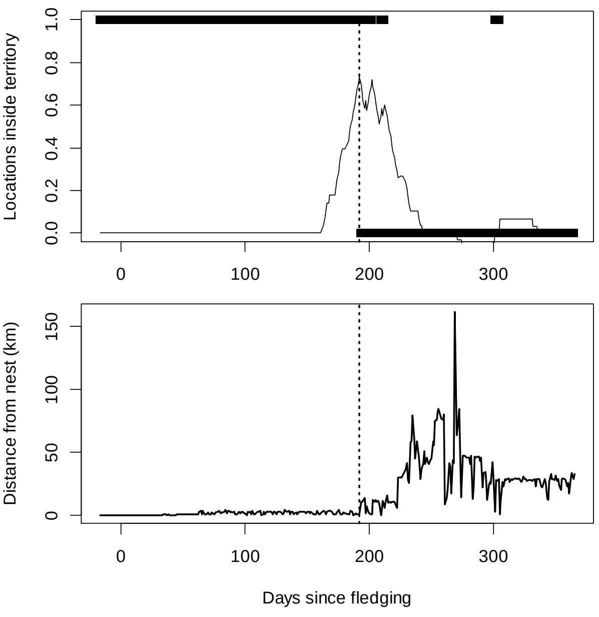

GE-437

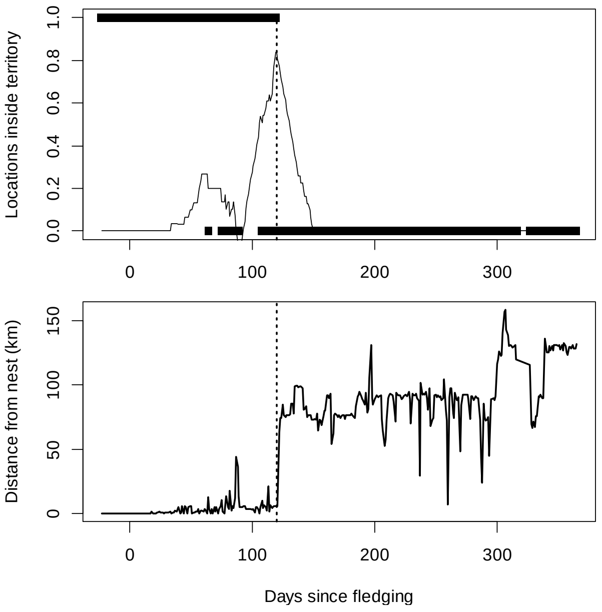

GE-440

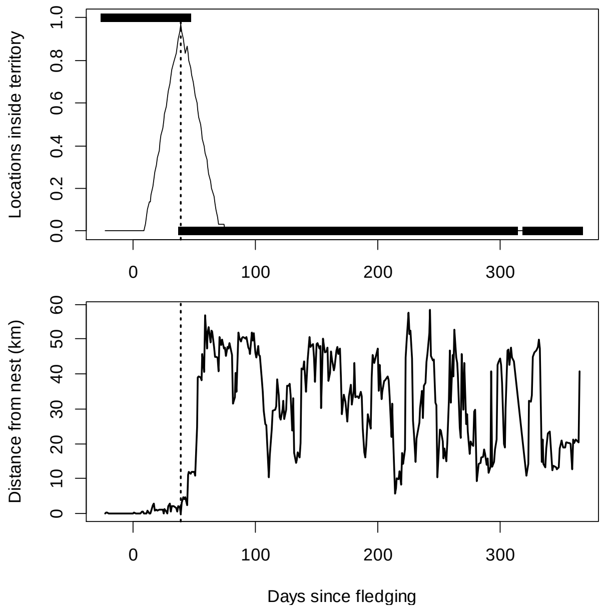

GE-441

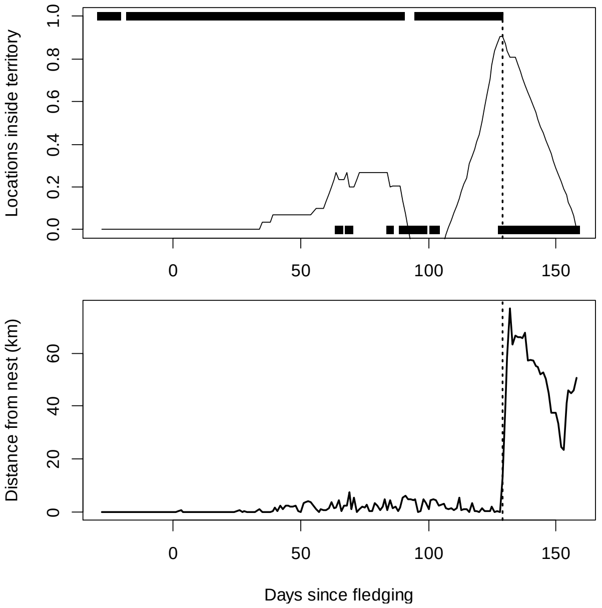

GE-442

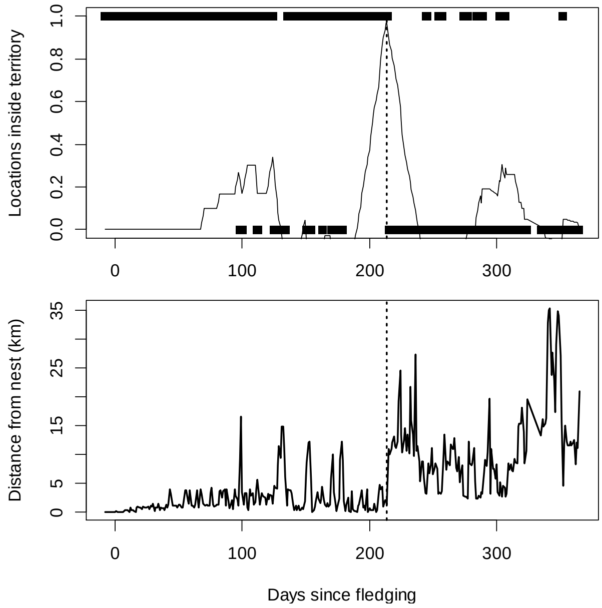

GE-856

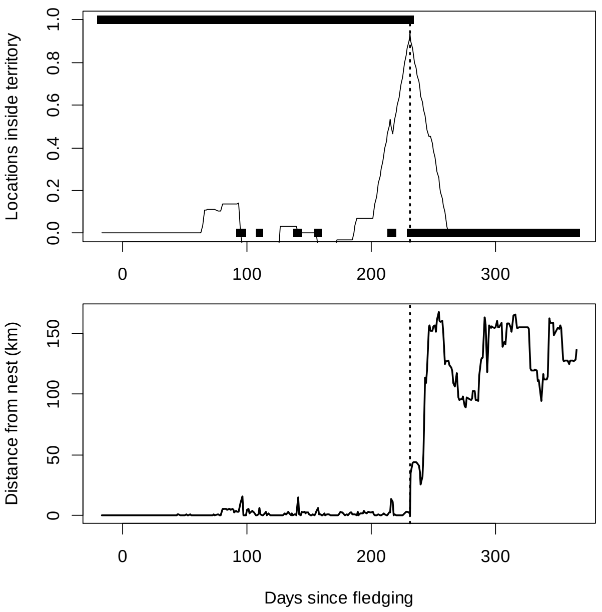

GE-304

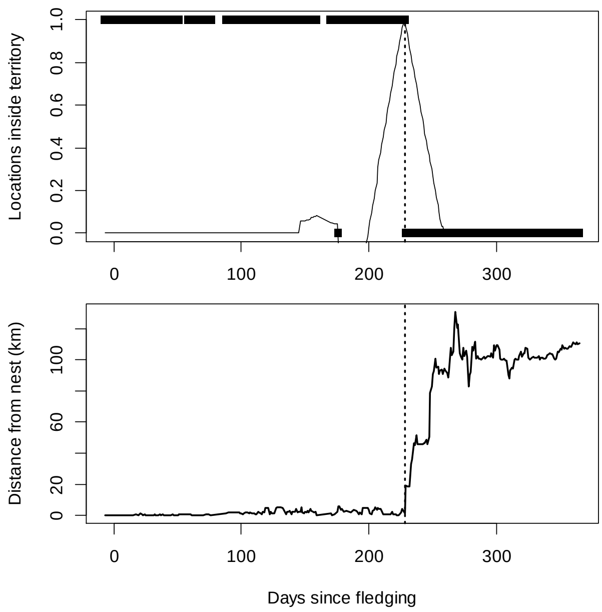

GE-109

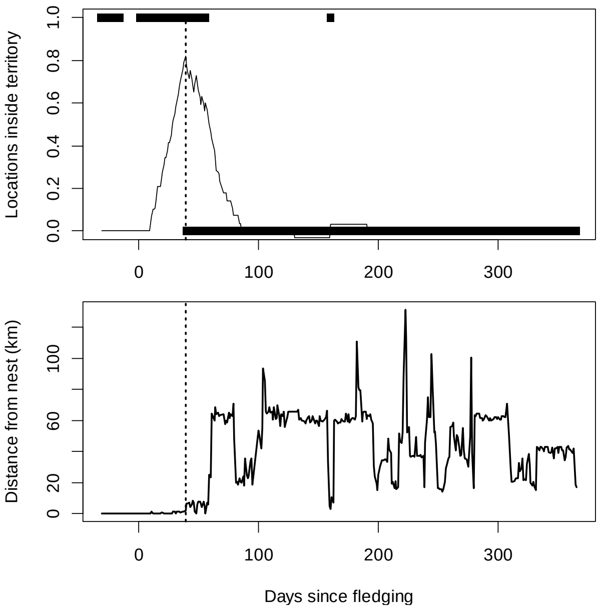

GE-111

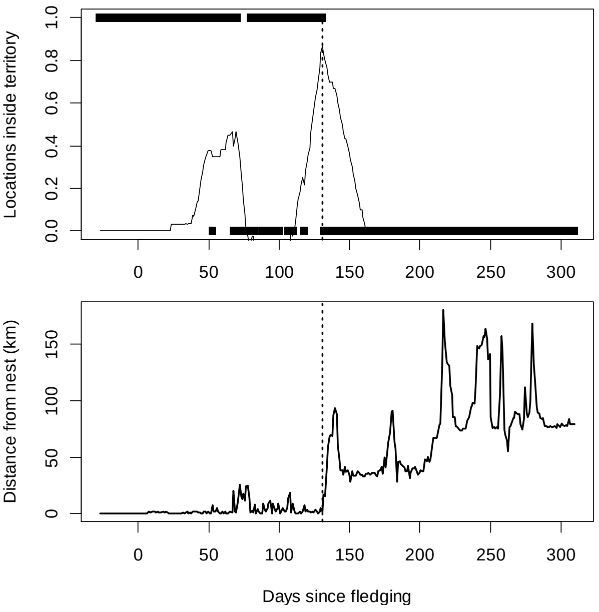

GE-382

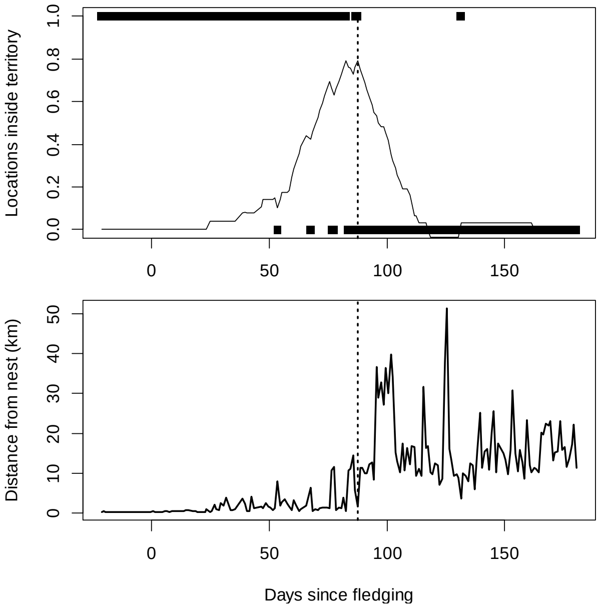

GE-115

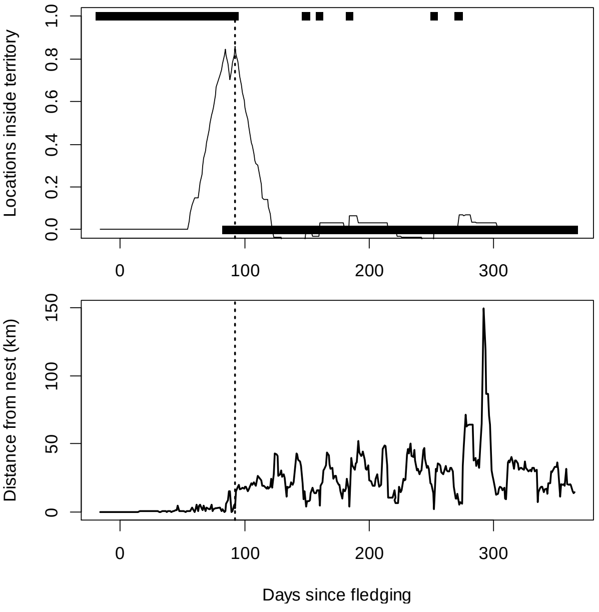

GE-124

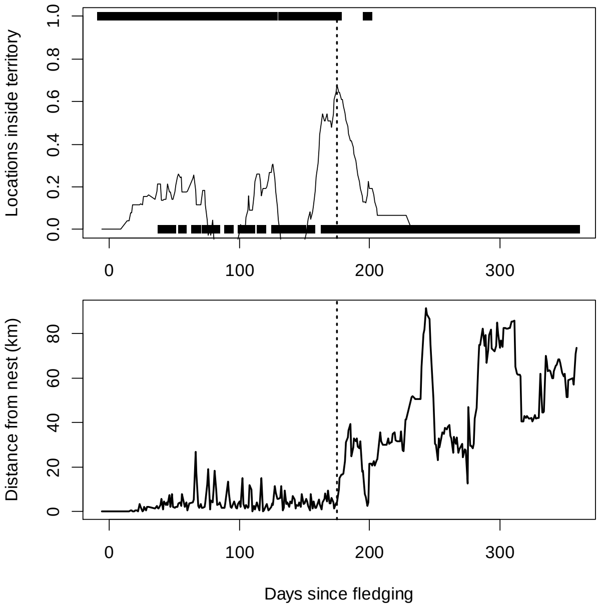

GE-139

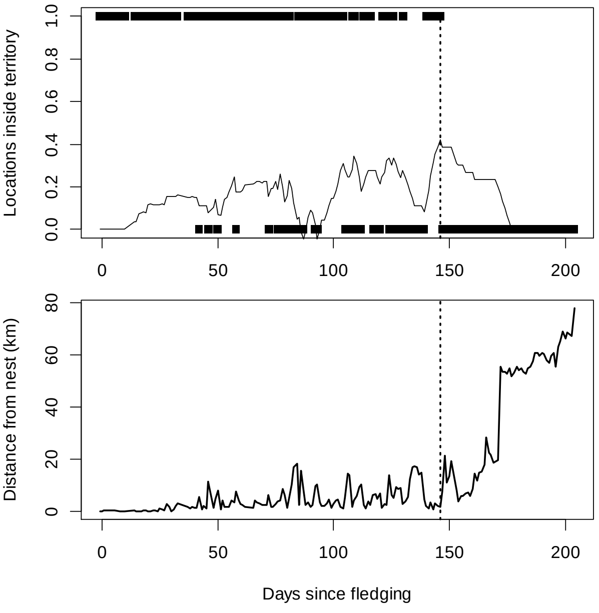

GE-142

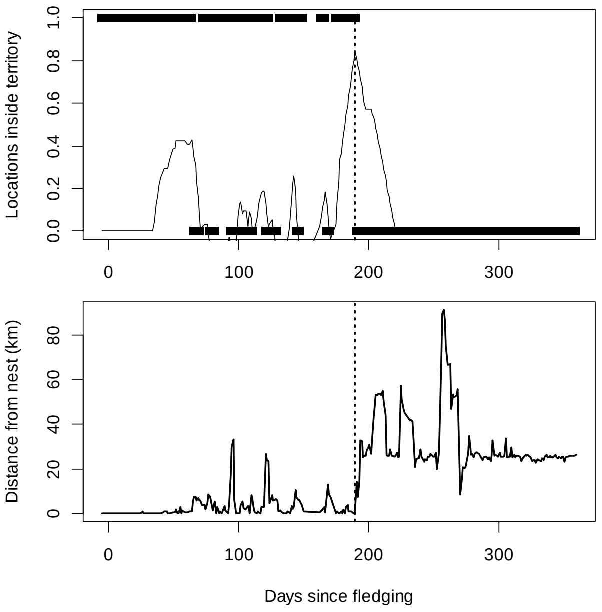

GE-857

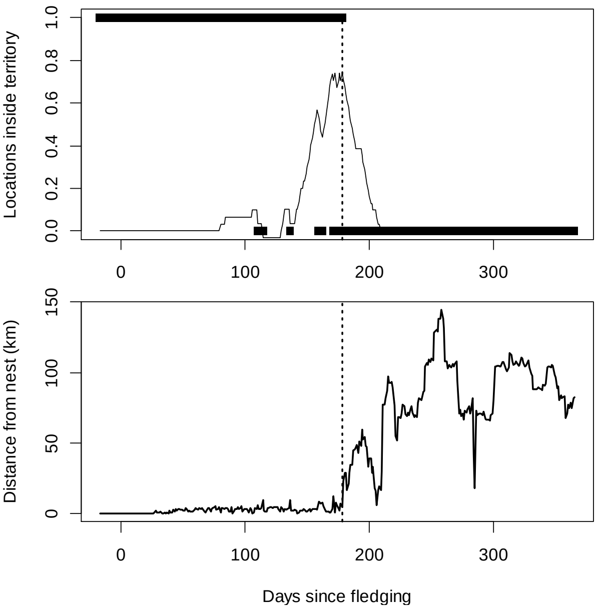

GE-858

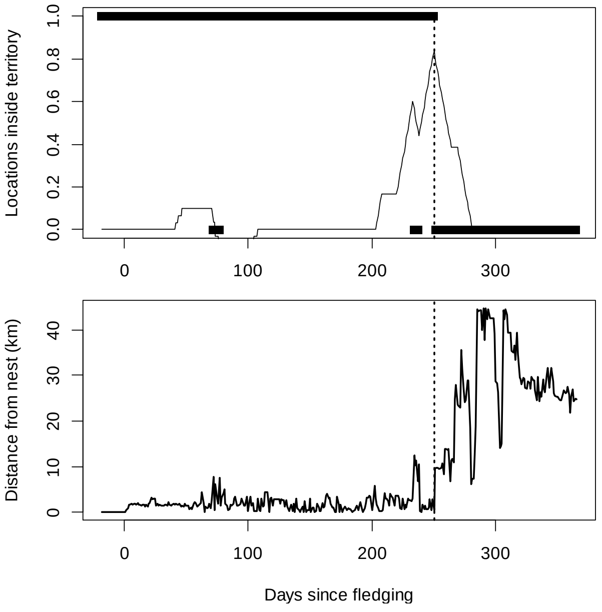

GE-106

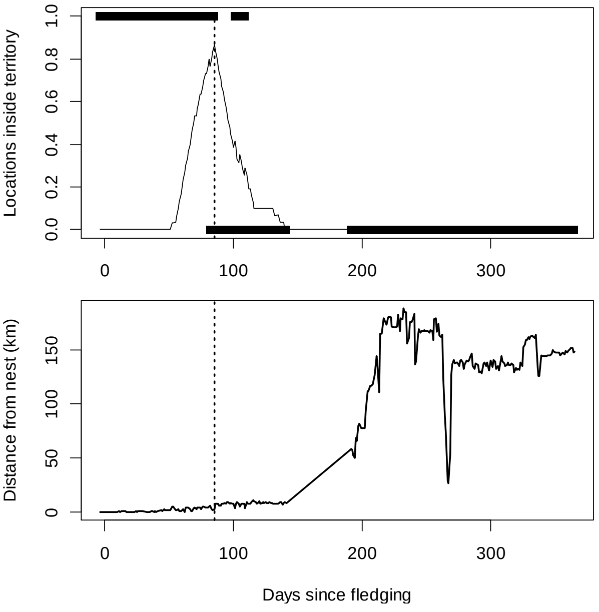

GE-107

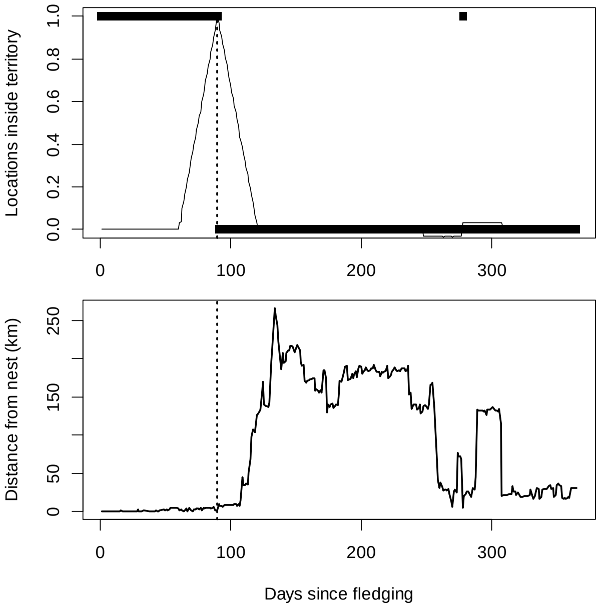

GE-867

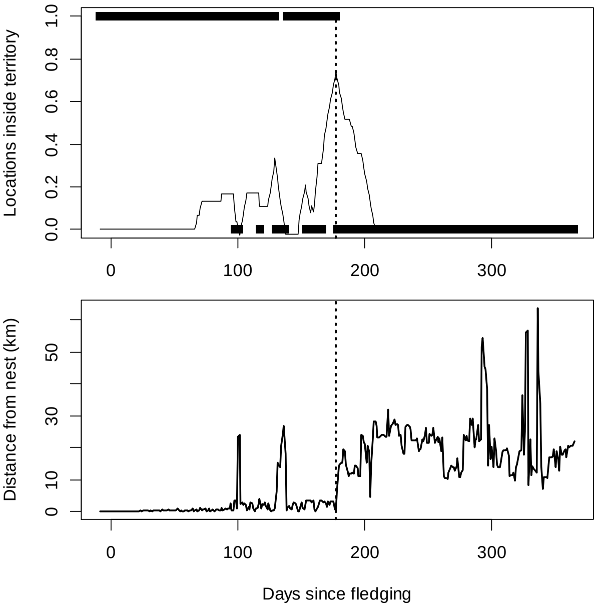

Supplementary figures. Estimates of the onset of dispersal for 24 golden eagles using method 12. Upper panel: points - occupation of natal home range (natal home ranges defines using the PAT model of golden eagle home ranges) at each time point (1 = in natal home range, 0 = outside of natal home range); solid line – ${}_{diff}P$ see Methods for details of calculation. Lower panel: solid line – distance from the nest.
